# Comparison of In-Person and Online Recordings in the Clinical Teleassessment of Speech Production: A Pilot Study

**DOI:** 10.3390/brainsci13020342

**Published:** 2023-02-17

**Authors:** Grégoire Python, Cyrielle Demierre, Marion Bourqui, Angelina Bourbon, Estelle Chardenon, Roland Trouville, Marina Laganaro, Cécile Fougeron

**Affiliations:** 1 Faculty of Psychology and Educational Sciences, University of Geneva, 1205 Geneva, Switzerland; 2 Neurorehabilitation Unit, Department of Clinical Neurosciences, CHUV, 1011 Lausanne, Switzerland; 3 Laboratoire de Phonétique et Phonologie, UMR 7018, CNRS/University Sorbonne Nouvelle, 75005 Paris, France

**Keywords:** speech and language therapy, teleassessment, telerehabilitation, motor speech disorders, speech and voice disorders, apraxia of speech, dysarthria, dysphonia, speech analysis, recording devices

## Abstract

In certain circumstances, speech and language therapy is proposed in telepractice as a practical alternative to in-person services. However, little is known about the minimum quality requirements of recordings in the teleassessment of motor speech disorders (MSD) utilizing validated tools. The aim here is to examine the comparability of offline analyses based on speech samples acquired from three sources: (1) in-person recordings with high quality material, serving as the baseline/gold standard; (2) in-person recordings with standard equipment; (3) online recordings from videoconferencing. Speech samples were recorded simultaneously from these three sources in fifteen neurotypical speakers performing a screening battery of MSD and analyzed by three speech and language therapists. Intersource and interrater agreements were estimated with intraclass correlation coefficients on seventeen perceptual and acoustic parameters. While the interrater agreement was excellent for most speech parameters, especially on high quality in-person recordings, it decreased in online recordings. The intersource agreement was excellent for speech rate and mean fundamental frequency measures when comparing high quality in-person recordings to the other conditions. The intersource agreement was poor for voice parameters, but also for perceptual measures of intelligibility and articulation. Clinicians who plan to teleassess MSD should adapt their recording setting to the parameters they want to reliably interpret.

## 1. Introduction

In speech and language therapy (SLT), telepractice has the potential to improve access to healthcare, for example, during severe pandemics, but also more generally for persons living in remote locations.

When speech production is teleassessed perceptually or acoustically by speech and language therapists (SLTs), one crucial aspect that can interfere with a reliable judgment of normality/alteration of speech is the amount of acoustic information lost or distorted due to the quality of the recording hardware or/and online transmission. In neurological diseases, remotely collecting speech samples of sufficient quality may have the potential to facilitate the diagnostic process and the access to speech-based biomarkers [1,2,3].

The aim of the present study is to estimate the reliability of telepractice when recording speech production online to compute perceptual and acoustic parameters.

### 1.1. The Assessment of Speech Production

In its broader sense, speech integrates a large array of dimensions, such as articulation, fluency, prosody, and voice. To assess and analyse speech, there are two main complementary approaches [4] in SLT clinical practice:

- Perceptual judgments, i.e., the clinician relies on her/his personal expertise to judge whether the perceived speech is unaltered or distorted, and if so, to which extent, usually by means of rating scales that can be filled in, either in front of the speaker or based on recordings;
- Acoustic measurements, i.e., the properties of the acoustic signal are quantified with the help of speech analysis software which are able to compute parameters that are not easily definable by ear, such as specific spectral components; various software is available and best practice recommendations/tutorials are only just emerging to compute acoustic measures for clinical purposes [5,6].

To assess speech production, for instance, in motor speech disorders (MSD), clinicians often rely on low-technology assessment tools with paper and pencils that focus on auditory–perceptual judgments [7,8]. MSD assessment batteries have traditionally been validated in person in quiet environments, such as soundproof research labs. However, in-person assessment might not be ideal or even possible in some situations, leaving online assessment the best (or only) option available to evaluate speech production.

### 1.2. The Teleassessment of Speech Production

In comparison to in-person examination, teleassessing speech production implies that the clinician and the examinee interact audio-visually by internet (on a smartphone, tablet, computer, television, connected by wired, satellite, or mobile networks). When the speech signal is transmitted from one person to another, it is compressed in packets which reduce its quality.

Nowadays, online speech compression algorithms better preserve the core parameters of speech, thanks to major improvements in the past years and to broadband high-speed internet. For example, the popular Zoom software (Zoom Video Communications, Inc., San Jose, CA, USA) prioritizes audio over video in low-bandwidth situations, and supports high-definition audio. However, it is frequently reported that speech quality still suffers from signal compression artifacts in online interactions [9,10]. For instance, it has been shown that fine-grained parameters used to assess voice quality such as shimmer, jitter and harmonic-to-noise ratio (HNR) were significantly distorted by online compression, regardless of the internet speed (ranging from 0.5 to 1.5 Mbit/s in [11]). More recently, it has been documented that recordings on Zoom contain sudden drops in intensity [12], shorten the duration of phonemes and more specifically fricatives [13], or stretch the spectral space of vowels [14], in comparison to in-person recordings made with various combinations of hardware and software.

Even if the examinees’ satisfaction about speech production teleassessment seems relatively high (e.g., >80% of satisfied examinees in [15,16]), large-scale studies evaluating teleassessment protocols in adults with rigorous experimental designs are still sparse [17]. In children, it has been suggested that teleassessing speech might be less reliable than teleassessing language [18,19], although a recent study showed that child speech intelligibility could be reliably measured on online video recordings with Skype, Google Hangouts, or FaceTime [20].

A systematic review on the use of telepractice (though not focusing on its reliability) suggested that teleassessment of MSD was feasible «despite some changes in the vocal signal wave after long-distance transmission» ([21], p. 6), without distinguishing between perceptual- and acoustic-based assessments. More particularly for the teleassessment of voice quality, recent guidelines [22] based on several research reports recommend using professional equipment to record voice samples. According to these guidelines, the quality of recordings stemming from the smartphone of the examinee at home is acceptable to compute the f0, but not jitter and shimmer. Subsequent studies also conclude that frequency components were the only voice parameters reliably computable in person via smartphones or tablets [23,24] (see, however, [25] for a more nuanced view regarding an acoustic voice quality index adequately computed by mobile devices). In addition, ambient noise seems to affect certain acoustic measures related to voice quality (e.g., HNR, CPP, spectral slope, vowel space) [5,26], though not especially f0 [27].

In addition to the fact that most recommendations were not based on empirical assessments of reliability, the methodological procedures to evaluate the reliability of teleassessments, i.e., the degree of agreement between in-person observations and online observations, differed across studies. Three methodological approaches are briefly presented below, the third approach being the one chosen for the present study.

### 1.3. The Reliability of Speech Production Teleassessments

First, the reliability of teleassessments can be estimated by comparing the **differential diagnosis accuracy** between real-time in-person assessment and teleassessment.

In such a procedure, one experimenter conducts the assessment and makes the diagnosis online by videoconference, whereas another experimenter simultaneously makes the diagnosis in person without interacting with the speaker. As two different raters are involved in the two conditions (one rater in person vs. another rater online), interrater variability represents a confounded variable in such an approach.

Wertz et al. [28,29] compared whether the two modalities led to the same differential diagnosis between broad categories of speech and/or language deficits (e.g., distinguishing between dysarthria and aphasia). These studies showed significant agreement between diagnoses made by telephone/television or in person.

Concerning the differential diagnosis of speech and voice impairments (e.g., dysarthria, apraxia of speech, dysphonia) and oromotor dysfunction, it was shown that teleassessments performed in 150 participants (82 with dysphonia, 37 with dysarthria, 19 with uncertain diagnoses, 8 with apraxia of speech sometimes associated with aphasia or dysarthria, 1 with psychogenic voice or speech disorder, 1 with aphasia, 1 with laryngectomy, 1 with stuttering) with specialized equipment and satellite-based connectivity provided sufficient information to make an accurate diagnosis for most participants (96%), and that the amount of uncertain diagnoses was not grossly higher in teleconference (19%) than in person (13%) [30].

When comparing the classification of dysarthria subtypes in 24 persons by means of perceptual and oromotor measures, agreement fell to 67% between in-person and online diagnoses made via professional software and hardware (e.g., two webcams mounted on a robotic arm) [16].

Second, the reliability of teleassessments can be estimated by comparing **perceptual ratings and/or acoustic measures** made in person vs. online, again with two different assessors.

The first studies of this approach by Hill et al. [31] and Theodoros et al. [32] are not detailed here because the in-person assessment and the teleassessment were conducted on different days, and ratings were essentially performed offline.

Subsequent studies [15,16] conducted a single assessment session, with two examiners rating simultaneously. One experimenter conducted the assessment and rated online by videoconference, whereas another experimenter simultaneously rated in person, but without interacting with the speaker. Experimenters were alternatively situated next to the participant or online, so that each experimenter was equally involved in both assessment conditions. Importantly, the distant experimenter did not rely only on the remote compressed speech signal to score speech parameters, but had also access to high-quality in-person audio and video recordings in order to check and correct the ratings offline when necessary.

Following such a procedure, speech and oromotor function were assessed in individuals with acquired dysarthria mostly due to stroke or traumatic brain injury [15] (n = 24) or neurodegenerative hypokinetic dysarthria [16] (n = 61). In both studies, poor agreement (generally interpreted with quadratic weighted Kappa < 0.6) was obtained for about half of perceptual/oromotor measures (such as parameters related to pitch, loudness, voice quality, facial musculature, and some aspects of intelligibility), whereas good agreement (generally interpreted with quadratic weighted Kappa > 0.8) was achieved for a minority of perceptual/oromotor measures (such as respiratory support, tongue movements and diadochokinetic rate). On top of perceptual measures, Constantinescu et al. [15] also performed three acoustic measures offline on high-quality recordings, namely sound pressure level (SPL), duration of sustained vowel, and pitch range. Sufficient agreement was reached for these three measures, according to predetermined clinical criteria set below the minimal changes expected after Lee Silverman Voice Treatment (e.g., ±3 s for the duration of sustained vowels) [33].

Third, the reliability of teleassessments can be estimated by comparing perceptual ratings and/or acoustic measures made on recordings collected in person vs. online, i.e., relying strictly on the compressed speech signal. Therefore, this third method focuses primarily on the **quality of the recorded speech signal** in everyday online communication.

This approach is particularly interesting to assess if standard material accessible to potential examinees at home could nowadays provide recordings of acceptable quality. A few years ago, Vogel et al. [34] examined the agreement on acoustic measures of neurotypical speakers’ speech samples captured from four sources: three devices recording simultaneously in person (hard disc recorder, laptop, and smartphone) in addition to an online condition over an analogic landline telephone. The agreement between recording sources was estimated by Spearman’s coefficients and by the proportion of root mean squared error (RMSE), i.e., the RMSE of each acoustic measure divided by the median observation across sources. When comparing the telephone condition to the others, poor reliability (i.e., RMSE > 10%) was reported for nine voice parameters (jitter, shimmer, NHR, voice turbulence, soft phonation, CPP, CPPs, SD of f0, HNR), leaving only the mean f0 as a reliable measure in telephone recordings. All timing measures (number of silences, mean silence duration, SD of silence duration, total silence time, total speech time, speech-silence ratio) were insufficiently reliable online over the telephone.

More recently, similar observations were reported in linguistic research for online Zoom recordings of word reading by a single neurotypical speaker, which permitted sufficiently reliable f0 calculation, but revealed inaccuracies in general for shimmer, jitter, and amplitude differences between the first and second harmonic (H1–H2) and formant values in particular for F2 [13]. As for auditory–perceptual ratings, they seem feasible on online recordings for certain characteristics of dysphonia: four parameters of voice (overall severity, roughness, breathiness, strain) showed good reliability/accuracy when rated on a scale from mild to moderate by expert clinicians listening to recordings of sustained vowels and passage reading played via Zoom or Cisco Webex [35].

Crucially, when comparing in-person recordings originating from different devices or audio formats [13,23,24,36,37], substantial discrepancies have been observed on consonant/vowel duration and voice measures (such as formants, HNR, SNR, shimmer, frics). Similarly, when different software was used to compute acoustic measures on high-quality in-person recordings, discrepancies also appeared in the values according to the software [12].

The Internet connection speed and associated fluctuations also influence the quality of online recordings [3,38]. An experiment simulating bandwidth constraints on the Opus codec used by Zoom for speech compression suggests that some acoustic measures (e.g., jitter, shimmer, HNR, CPP) derived from dysarthric speech recordings especially suffer from low-bandwidth situations [39].

In sum, most studies interested in the reliability of speech teleassessment have compared the ratings/measures of two different assessors in terms of either differential diagnosis (see first method above) or by sending high-quality in-person recordings to the remote assessor (see second method above). In both cases, custom-built calibrated software and professional hardware were used. However, these recording conditions do not reflect the equipment situation of actual speech-impaired individuals who do not own professional material at home. Moreover, most clinical studies included only perceptual measurements, although recent professional guidelines about teleassessment of dysarthria recommend performing not only perceptual, but also acoustic measures online [40].

Critically, recent studies conducted on neurotypical speakers warned that Zoom recordings could distort the speech signal in terms of duration and intensity for basic tasks such as sustained vowels [12] or word reading [13].

To our knowledge, no study on the clinical teleassessment of speech used the third method to examine agreement between measures (both perceptual and acoustic) performed on in-person recordings obtained by different devices vs on recordings generated by online videoconferencing.

### 1.4. Purpose of the Study

The aim of the present study is to investigate which speech parameters can be reliably interpreted in recordings collected without professional equipment. More specifically, we compared two teleassessment recording conditions to a baseline/gold standard high-quality in-person recording condition. Simultaneous recordings of a screening battery for MSD were collected from different sources in order to explore if standard equipment (laptop built-in microphone) or/and a popular videoconference platform (Zoom; Zoom Video Communications, Inc., San Jose, CA, USA) could provide speech samples of sufficient quality for the teleassessment of speech production, or if high-quality in-person recordings remain indispensable in clinical practice.

## 2. Materials and Methods

### 2.1. Participants

Fifteen neurotypical native French speakers took part in this experiment. In order to cover a variety of speech signals, people from different ages (25–83, mean age 53), different genders (ten women, five men) and with different regional accents (France, Switzerland) were included (Table 1). They were recruited in Switzerland and in France as part of a larger study between May and August 2020. They gave written informed consent in accordance with the Declaration of Helsinki, which was validated by the local ethical research committee (CCER Geneva protocol code 2018-00212). The participants had no history of neurological or psychiatric disorders. Speech production was unimpaired in the present sample of neurotypical participants, as no speaker reached the cut-off score for a diagnosis of MSD in the MonPaGe-2.0.s screening protocol (i.e., total deviance scores < 2) [41,42].

### 2.2. Materials and Procedure

Speech production was evaluated with several modules of the MonPaGe-2.0.s screening protocol [41,42], a computer-based assessment tool of MSD in French (freely available here: https://lpp.in2p3.fr/monpage/, accessed on 15 April 2020). This screening battery records audio files elicited by different speech production tasks for further guided perceptual and acoustic analyses (see below). The assessment session lasted 30–45 min.

**Figure 1:**
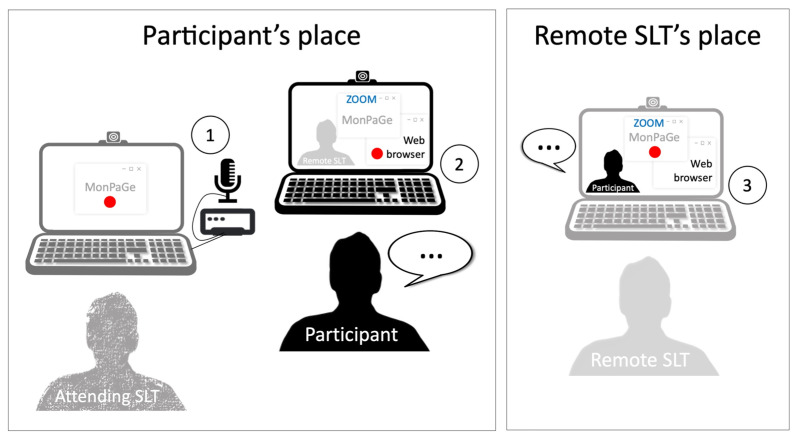
Setting for the simultaneous recording from three sources, namely (1) high-quality in-person hardware (local HQ condition), (2) in-person standard hardware, with software automatically sending audio files online (local SQ condition), and (3) remote standard hardware (online condition).

Recordings of the participants’ speech were simultaneously collected from three sources (Figure 1) in order to evaluate intersource agreement, i.e., to which extent recordings captured by devices of different quality or situated in different locations could provide similar perceptual and acoustic measurements. The three recording settings were as follows (see also Table 2 for a summary of key differences between these conditions):

1. **Local high-quality condition (local HQ):** Speech was recorded at the participant’s home on a Dell laptop running MonPaGe-2.0.s. The in-person recording source was a professional headset condenser microphone (Shure SM35-XLR) connected to an external USB sound card (Scarlett 2i4 Focusrite). This condition served as the “gold standard” condition, and was compared to the two teleassessment settings below;
2. **Teleassessment with local standard quality recordings (local SQ):** Speech was recorded at the participant’s home on an Apple laptop running Zoom and a website in Safari allowing continuous recording. The in-person recording source was therefore the built-in microphone of the laptop. The website used the recorder js library by Matt Diamond (https://github.com/mattdiamond/recorderjs, accessed on 22 April 2020) to access the laptop’s microphone via the browser and to record audio. The recorded audio was automatically sent to a server via ajax once the recording was finished and was therefore easily available to the remote assessor. Note that this setting resembles previous linguistic research approaches using both Zoom and the smartphone of the participant as a recorder with a specialized app transmitting the audio files to the remote experimenter (e.g., [43]); however, here the same device (laptop) was used both to conduct the Zoom session and to record the participant;
3. **Teleassessment with remote zoom recordings (online):** Speech was recorded at the remote experimenter’s place on an Apple laptop running MonPaGe-2.0.s and sharing the screen on Zoom. The remote recording source was therefore the built-in microphone of the laptop; the speech signal of the participant was played by the built-in speakers of the same laptop via Zoom.

#### 2.2.1. Recording Setting

For most participants (11 out of 15), two experimenters were involved. The first experimenter managed the local recordings at the home of the participants on two different computers (one for the local HQ condition, the other one for the local SQ condition), without helping the participants in any way throughout the assessment (Figure 1, left). The remote experimenter delivered MonPaGe-2.0.s in fullscreen mode via Zoom Client 5.0.1 with a Zoom education account (Figure 1, right). Experimenters were alternatively situated next to the participant or online, so that each experimenter was equally involved in both assessment conditions. The four remaining participants managed the two local computers themselves, as they were already familiar with the MonPaGe-2.0.s protocol. Microphone volume (input) and speaker volume (output) were set to 100%. As the audio files from the three recording sources were all in wav format (44.1 KHz, 16 bits), they were used for the analysis without any conversion.

The bandwidth (upload and download speeds at the participant’s home and at the SLT’s office) was measured with the iOS app SpeedTest.Net 4.2.1 (https://www.speedtest.net/apps, accessed several times between 11 May 2020 and 19 August 2020) at the beginning of the assessment, after half of the tasks, and again at the end of the assessment. The high-speed internet connection in both locations was mainly generated by 4G mobile networks in Switzerland or in France.

#### 2.2.2. Perceptual and Acoustic Analyses

All analyses were performed on the recordings, after the assessment, by three SLT judges/raters in order to evaluate the agreement among raters in addition to the agreement among recording sources. The three raters began with the recordings of the 15 participants from the online condition, then the local SQ condition, and finally the local HQ condition. For each condition, the 15 participants were analysed in random order.

Analyses of the recordings were guided in PRAAT 6.1.09 (https://praat.org, accessed on 4 May 2020) by means of a script allowing the manual adjustment of the boundaries encompassing the target speech samples on which acoustic parameters were then computed (see below). In MonPaGe-2.0.s, acoustic measures are computed semi-automatically: it is still up to the clinician to subjectively select and segment the speech samples included in the computation of acoustic speech parameters, and to control for the plausibility of the output scores. Because the speech signal quality could differ according to the recording condition, SLT raters had to delimit the boundaries of the speech samples manually for each recording condition independently, instead of applying the exact same boundaries/time intervals to the three recording conditions. It was thus possible that the speech samples selected for the analyses of a given parameter did not have exactly the same duration in each condition. If the signal was distorted due to the recording device quality or/and online compression, it was hypothesized that putting boundaries on local SQ recordings or/and online recordings would be less precise and would lead to more divergence with local HQ recordings.

#### 2.2.3. Tasks and Speech Parameters

Two **perceptual parameters** were considered in two different tasks of the MonPaGe-2.0.s protocol (see detailed description and list of stimuli in [41,42]):

1. Intelligibility: In this task, 15 target words randomly extracted from a dataset of 437 words, all having at least one phonological neighbour, appeared successively alongside their corresponding picture on a grid of colored shapes visible only to the speakers but not to the experimenter. Speakers had to instruct the experimenter where to place the target word in the grid by using a pre-defined sentence such as “Put the word *leaf* (=target word) in the red square”. In the original MonPaGe-2.0.s protocol, assessors transcribe the words and place them on the mentioned locations in real-time, i.e., during the assessment when interacting with the speakers. Here, the three SLT raters performed the same task but offline, based on the audio recordings captured during the assessment. Intelligibility was rated perceptually as the **number of words incorrectly understood** by the SLT raters out of the actual 15 target words speakers had to produce. In total, 675 target words (15 items × 15 participants × 3 conditions) were transcribed by each SLT rater;
2. Articulation in pseudowords: In this task, 53 pseudowords containing all French phonemes were presented both orally and in written form. The pseudowords followed the phonotactic restrictions of French. Speakers had to repeat or read aloud each pseudoword. Recordings were rated offline in MonPaGe-2.0.s through a guided perceptual procedure: SLT raters could play the recorded answers as needed, and were asked to judge the production of 151 targeted sounds/sequences of sounds (e.g., /r/, /f/ and /o/ in *rafo* or /v/ and /str/ in *vastra*) as correctly produced, incorrectly produced or containing a sound/recording problem. Articulation was rated as the **number of errors** perceived on the 151 target phonemes or target syllables. In total, 6795 target sounds (151 items × 15 participants × 3 conditions) were coded by each SLT rater.

Fifteen **acoustic parameters** were computed on the recordings of several speech samples elicited in different tasks (see detailed description and list of stimuli in [41,42]):

1. Five parameters related to *speech rate* were measured in three different tasks:
  - Repetition of the days of the week: In this task, speakers had to repeat the overlearned series of the days of the week continuously for at least 30 s. SLT raters had to manually label the onset of the production on the recordings, and the PRAAT script automatically defined a window of 30 s from this onset (which could be manually adjusted to the right if needed in order to not cut a word). The rate over this window was computed as the number of words produced divided by the window duration (**Rate_Days in words per second**);
  - Sentence reading: In this task, speakers had to read a short sentence containing seven CV syllables. SLT raters had to manually label the onset and offset of the sentence on the recordings, and speech rate was automatically computed as the number of syllables (7) divided by the sentence duration (**Rate_Sentence in syllables per second**);
  - Diadochokinesia (DDK): In this task, speakers had to repeat as fast and as precisely as possible CV or CCV syllables (/ba/, /de/, /go/, /kla/ or /tra/; alternative motion rate—AMR) and a sequence of three CV syllables (/badego/; sequential motion rate—SMR) for at least 4 s. SLT raters had to manually label the onset of the production on the recordings, and the PRAAT script automatically defined a window of 4 s from this onset (which could be manually adjusted to the right in order to not cut a syllable, if needed). The DDK rate was computed as the number of syllables produced divided by the window duration (**Rate_DDK AMR CV, AMR CCV and SMR CV in syllables per second**).
2. Eight parameters related to *voice* were measured in two different tasks:
  - Sustained /a/: In this task, speakers had to maintain the vowel /a/ for at least 2 s at a comfortable pitch and loudness. SLT raters had to manually label the onset of the /a/ and the PRAAT script automatically defined a window of 2 s from the onset. Raters were instructed to move this 2 s window and/or to tune the frequency range to insure adequate f0 detection. Over this window, five voice measures were automatically computed: **Jitter (5-point Period Perturbation Quotient), Shimmer (11-point Amplitude Perturbation Quotient), HNR, smooth Cepstral Peak Prominence (CPPs), f0 standard deviation**;
  - Sentence reading: On the segmented sentence used for the speech rate measure (see above), three measures related to voice and speaking f0 were also computed as follows: **mean f0, f0 standard deviation, CPPs**.
3. Maximal phonation time: In this task, speakers had to maintain voicing on the vowel /a/ as long as possible in a single breath at a comfortable pitch and loudness in two trials. SLT raters had to segment the recordings, and the duration of the longest trial was retained as a measure of **Maximal Phonation Time in seconds**.
4. Prosody: In this task, speakers had to read aloud a four syllable sentence the first time with affirmative prosody and the second time with interrogative prosody. In order to measure the linguistic prosodic contrast between the two sentences, SLT raters had to manually label the onset and the offset of the sentence on the recordings, and to adjust f0 detection. The difference in f0 modulation (f0 range in semitones) between the beginning and the end of the sentences served to compute the **Prosodic Contrast in semitones**.

#### 2.2.4. Reliability Analyses

Two agreement measures were considered as follows:

1. **Intersource agreement**: The agreement of the scoring between the recording sources was calculated in two-by-two comparisons between raw scores of each rater for each participant stemming from local HQ vs. online recordings and from local HQ vs. local SQ recordings. For both teleassessment settings (local SQ and online), the local HQ condition served as a gold-standard baseline condition. Intersource differences were calculated on absolute values for each speech parameter of each participant scored by each rater to control for differences going in opposite directions (e.g., f0 computed higher online than in person for a given participant or by a given rater, but lower for another participant or by another rater). A ratio of divergence among sources was calculated by dividing the absolute difference between the local HQ and the conditions of interest (|local HQ *−* online| or |local HQ − local SQ|) by the value of the local HQ condition considered as the reference (|local HQ *−* online|/local HQ or |local HQ − local SQ|/local HQ). For example, a maximal phonation time (MPT) of 18 s according to local HQ recordings compared to an online MPT of 18 s would give 0 source divergence (i.e., |18 **−** 18|/18 = 0 or 0% if expressed as a percentage), whereas an online MPT of 12 s would give 0.33 source divergence (i.e., |18 − 12|/18 = 0.33 or 33% if expressed as a percentage). For each speech parameter, a mean **percentage of source divergence** across participants and raters was then calculated.
2. **Interrater agreement:** The agreement of the scoring between the three SLT raters was also examined, primarily as a control measure, but also to test to what extent recordings of lower quality increased interrater variations as compared to high-quality recordings. For each speech parameter in each participant, the averaged scoring of the three SLT raters served as a reference value (SLT1 + SLT2 + SLT3/3 = meanSLT). Interrater differences were calculated on absolute values between the scoring of each rater and the mean scoring (|SLT1 − meanSLT|, |SLT2 − meanSLT| and |SLT3 − meanSLT|), and each of these differences was divided by the reference value (|SLT1 − meanSLT|/meanSLT, |SLT2 − meanSLT|/meanSLT and |SLT3 − meanSLT|/meanSLT). The three obtained ratios were finally averaged to compute a mean ratio of divergence among raters. For example, for the prosodic contrast, if SLT1 scored 5 semitones for a given participant, but SLT2 scored 9 semitones and SLT3 10 semitones, the averaged scoring would be 8 semitones for this participant ((5 + 9 + 10)/3). In this case, the mean rater divergence would reach 0.25 (i.e., (|5 − 8|/8 + |9 − 8|/8 + |10 − 8|/8)/3 = 0.25 or 25% if expressed as a percentage). For each speech parameter, a mean **percentage of rater divergence** across participants was then calculated.

#### 2.2.5. Statistical Analyses

Percentages of divergence among sources and among raters were compared with paired *t*-tests. Moreover, intersource agreement on raw scores was estimated by intraclass correlation coefficients (ICC) considering two-way random effects, absolute agreement, and single measurement [44]. Two by two comparisons were carried out independently of the raters with the local HQ condition always serving as baseline reference. As a control measure, but also to estimate the impact of recording quality, ICCs were computed between the scoring of the three SLT raters in each condition. ICC values were interpreted according to [45], i.e., poor reliability under 0.5, moderate reliability between 0.5 and 0.75, good reliability between 0.75 and 0.9, and excellent reliability above 0.9. Analyses were performed with IBM^®^ SPSS Statistics 26 software.

Finally, in order to estimate whether the scoring differences between the online condition and the local HQ condition varied according to the internet connection speed, Pearson correlations were computed for each speech parameter between the percentage of divergence among recording sources (online vs. local HQ) and the mean broadband speed for each bandwidth parameter (upload and download speeds at the participant’s home, upload and download speeds at the SLT’s location averaged across the beginning, the middle and the end of the assessment). Only significant negative correlations were considered, i.e., slower broadband speed leading to greater divergence.

## 3. Results

Raw scores are available in the Supplementary Materials. For all acoustic and perceptual measures, mean raw differences and mean ratios of divergence are reported in Table 3 for intersource data and in Table 4 for interrater data. Percentages of divergence were highly variable according to the speech parameters. **Intersource** divergence (Table 3) was overall larger when comparing local HQ recordings to online recordings (mean 53.8% across parameters) rather than to local SQ recordings (mean 25.2% across parameters) (*p* = 0.05). **Interrater** divergence (Table 4) was overall larger when ratings were computed on online recordings (mean 9.3% across parameters) vs. on local HQ recordings (mean 6.1% across parameters) (*p* = 0.04), but other comparisons failed to reached significance (local HQ vs. local SQ: *p* = 0.28; local SQ vs. online: *p* = 0.30). ICCs for intersource agreement will be presented first followed by ICCs for interrater agreement.

### 3.1. Intersource Agreement

The agreement between measures based on recordings from local HQ vs. **online** conditions varied greatly according to the type of speech parameters, with intersource ICC reaching 0.56 on average and ranging from 0.08 to 0.99 (Table 5). For perceptual measures, the ICC indicated poor reliability in detecting distortions on pseudowords (0.47) as well as in identifying words within a non-constraining context in the intelligibility task (0.27). For most acoustic measures related to speech rate, excellent reliability (ICC > 0.9) was achieved, except for sentence reading, in which values fell just below this threshold (0.87). For the prosodic contrast and most voice parameters, poor reliability (ICC < 0.5) was observed, except the mean f0 computed from sentence reading, which showed excellent reliability (0.99). The MPT led to good reliability (0.88).

The agreement between measures based on recordings from local HQ vs. **local SQ** conditions was also highly variable depending on the type of speech parameters, with intersource ICCs ranging from 0.17 to 1 and reaching 0.70 on average (Table 5). For perceptual scores, the ICC indicated moderate reliability in detecting distortions on pseudowords (0.69) and poor reliability on the number of words correctly identified in the intelligibility task (0.35). For most speech rate parameters, excellent reliability (ICC > 0.9) was achieved, except for sentence reading, with values falling just below this threshold (0.89). For all voice parameters, moderate to poor reliability (ICC < 0.75) was observed, except the mean f0 computed from sentence reading showing excellent reliability (0.99). Prosodic contrast showed good reliability (0.76), and MPT showed excellent reliability (0.99).

### 3.2. Interrater Agreement

The ICC between the three SLT raters reached 0.91 on average for local HQ recordings, 0.84 for local SQ recordings, and 0.82 for online recordings (Table 5). In each condition, ICCs were very variable depending on the speech parameters ranging from 0.13 to 1 (Table 5). For the perceptual measure of articulation (error detection in pseudowords), the ICC indicated excellent interrater reliability only on local HQ recordings (0.98), but moderate to good reliability in the lower quality conditions (0.64 for local SQ recordings and 0.76 for online recordings). For the perceptual measure of intelligibility (word identification), poor interrater reliability was observed in all recordings conditions (0.13 to 0.46). All speech rate parameters and MPT showed excellent interrater reliability in all conditions (ICC > 0.9), except the values for MPT fell just below this threshold in the online condition (0.88) and the sentence reading rate led to good reliability in local SQ and online conditions (0.77). For voice parameters, excellent reliability (≥0.9) was observed for CPPs, HNR, shimmer and mean f0 in all conditions. The jitter computation led to excellent interrater reliability only on local HQ recordings (0.93), but to good reliability in the other conditions (0.85 for local SQ recordings and 0.79 for online recordings). The standard deviation of f0 led to moderate/poor reliability in all conditions (<0.75), except for its computation on sentence reading in the local SQ condition which showed excellent interrater reliability (0.95). For the prosodic contrast, good reliability was observed across raters only in the local HQ condition (0.79), but the reliability was moderate to poor in other conditions (0.60 for local SQ recordings and 0.43 for online recordings).

### 3.3. Impact of Internet Speed

Internet connection offered variable down/upload speeds, ranging from 0.4 to 187.7 Mbps. On average, the download and upload speeds reached, respectively, 33.8 Mbps (SD = 25.7) and 9.2 Mbps (SD = 9.3) at the participant’s home and 47.8 Mbps (SD = 39.4) and 20.9 Mbps (SD = 13.0) at the SLT’s location, corresponding to current broadband high-speed internet standards. No significant negative correlation was found between the broadband speed and the percentage of divergence (all r*s* comprised between −0.28 and 0).

## 4. Discussion

The present experiment showed that it was feasible to administer a MSD assessment battery (here MonPaGe-2-0.s) in a teleassessment setting. Broadband speed did not play a major role when using high-speed internet: high ICCs were obtained for several tasks despite very heterogeneous speeds, and no significant negative correlation was found between internet speed and divergences between online vs local measures.

Crucially, however, only certain speech parameters could be reliably interpreted when compared to the gold standard HQ in-person condition, with increased reliability of the hybrid setting with in-person recordings transferred to an online server (the local SQ condition) compared to online recordings via Zoom. This observation is overall consistent with recent research reports recommending in-person recordings rather than online recordings for better audio signal fidelity [12,13,36]. However, even with such a hybrid approach, the two perceptual measures (intelligibility and articulation) and some acoustic measures (particularly for voice and prosody) led to substantial discrepancies when compared to local HQ recordings. Although interrater reliability was very high in the local HQ condition for most parameters, it remained poor for measures related to f0 variation and to intelligibility even in this gold standard condition, preventing any strong interpretation about these parameters. In the following, speech parameters will be discussed in more detail.

### 4.1. Perceptual Measures

Intelligibility scoring led to poor intersource and interrater agreement, as indicated by the low ICCs. As neurotypical speakers were tested here, a ceiling effect was expected in the two perceptual tasks. However, the maximal score of intelligibility (i.e., 15/15 words correctly identified by SLTs) was not reached by all participants in all conditions. In online recordings, up to three words (out of 15) per participant were incorrectly identified, whereas the two conditions with in-person recordings (SQ and HQ) led to a maximum of one misunderstanding per participant. These perceptual differences between online and local recordings could be due to the speech signal quality distorted by online compression. Speech samples of lower quality probably decreased the accuracy of word identification in sentences without contextual cues in such a way that phonological neighbours were confused by the listeners. In the current intelligibility task, the target words were unpredictable, and the scoring consisted of checking if the transcribed words were the actual targets that the participant saw on his/her screen. As the transcription of target words was made offline without lip reading contrary to the original MonPaGe-2.0.s protocol, further interpretations about the low reliability of this task should remain restricted to similar situations where lip reading is impossible (e.g., wearing a face mask or talking on the telephone). Interestingly, there was at least as much variation between raters as between recording conditions for intelligibility ratings. It is thus probable that intelligibility especially decreased online due to lower sound quality, but interindividual factors could also play a role in word identification. Note that poor agreement between online and in-person intelligibility assessments in speech-impaired individuals have also been previously reported either at the conversational level [16] or at the single-word level [15].

To identify articulation errors in pseudowords, intersource reliability was moderate for local SQ recordings and poor for online recordings as compared to local HQ recordings. As for interrater reliability, it was excellent only on local HQ recordings, but not on lower quality recordings (local SQ and online). Taken altogether, these perceptual differences could be due to speech signal distortions caused not only by online compression, but also by the quality of the in-person recording hardware. As neurotypical speakers were tested here, no error was perceived by the SLT raters in most participants in the local HQ recordings, whereas the same participants were considered as producing a few “errors” in local SQ and online recordings. In other words, signal distortions were sometimes considered as speech errors from the speakers.

When testing speech-impaired individuals online by means of perceptual measures, one should be careful not to over-diagnose and restrict the conclusions about intelligibility and articulation to the actual assessment setting: someone considered mildly unintelligible or producing minor articulation distortions on pseudowords when assessed online could indeed be judged as totally intelligible when tested in person in a quiet environment. As intelligibility and articulation measures do not seem to be comparable in person and online, it could be clinically relevant to assess these parameters not only in the office, but also systematically online, as many interactions take place online nowadays, and differences in sublexical and word perception by the listener may occur. Normative data of online intelligibility and articulation should ideally be developed in future research.

The perceptual measures in MonPaGe-2-0.s consist in transcribing words and identifying phonemes in pseudowords (resulting in binary scores), but other commonly used perceptual measures consist in filling in ordinal scales relative to the severity of the impairment (on a continuum). It is therefore possible that reliability estimates were more conservative here due to the binary nature of the data, as compared to continuous data, potentially explaining why some studies using rating scales reported reliable online measurements for some perceptual parameters [15,16,35].

### 4.2. Speech Rate Measures

As indicated by the high ICCs between local HQ and online measurements, online speech samples were of sufficient quality to calculate speech rate in words per second when repeating the days of the week and in syllables per second in DDK (both alternative and sequential motion rates). Even higher ICCs were found between local SQ and online measurements, suggesting that reliability could be further increased by using standard quality in-person recordings to measure speech rate. Concerning the reliability of speech rate measures computed in sentence reading, it was only good and not excellent in online and local SQ conditions as compared to the local HQ condition, probably because boundaries of the sentence under consideration were more precisely identifiable in the local HQ recordings. This interpretation is in line with interrater ICC being excellent only on local HQ recordings, but not in lower quality recordings (local SQ and online). Indeed, there was overall more divergence among raters when segmenting sentences in all conditions (2.3 to 7.1%) than when segmenting syllables in DDK or words in overlearned series repetition (0.4 to 1.2%). Nonetheless, as the recordings from the different conditions were sequentially treated by the SLT raters, it is not possible to exclude that a certain amount of reduced intersource reliability is partly related to intrarater variability, i.e., slightly different ways of segmenting the signal without the influence of the recording condition.

### 4.3. Voice Measures

High ICCs between local HQ and online measurements indicate that mean f0 calculation in online sentence reading was highly reliable, as already mentioned in previous reports [13,34]. However, to reliably compute other voice parameters (CPPs, HNR, shimmer, jitter), the present data confirm that high-quality in-person recordings remain indispensable. It is worth noting that interrater reliability was high for all voice parameters (interrater ICCs showing good to excellent reliability in all conditions), except for the standard deviation of f0. As it is possible that the three SLTs involved in this experiment lacked training about the computation of f0 variation and the verification of the plausibility of the scores in PRAAT, future training courses offered to MonPaGe-2.0.s users should particularly emphasise this aspect to neophytes. For such a feature, operationalization of rating procedures could likely improve interrater reliability. More generally, tutorials on accurate acoustic measurements for use by SLTs are still sparse [5], and should also be developed specifically for the teleassessment of MSD [40].

### 4.4. Maximal Phonation Time

Intersource ICC values concerning the MPT suggested that this duration measure was reliably computed on local SQ recordings, though not on online recordings. When recorded online via Zoom, sustained vowels actually suffered from signal distortion after several seconds in some participants and could be drastically underestimated (up to 17 s difference with HQ recordings, mean divergence of 12.5%); it was less the case in local SQ recordings (up to 8 s difference with HQ recordings, mean divergence of 1.7%). In line with the lower interrater ICC observed specifically in online vs. in-person recordings, these differences could be due to the speech signal quality distorted by online compression, but also to some extent to particular Zoom features in processing long sustained sounds (e.g., noise cancellation). Therefore, MPT should ideally be estimated only on local recordings, while not imperatively using professional quality recording hardware.

### 4.5. Prosodic Constrast

According to the ICC values, prosodic contrast was not reliably computed on online recordings, but computed fairly well on local SQ recordings, considering that a certain amount of interrater variability was also present for this parameter. Similarly to speech rate in sentence reading, the computation of the prosodic contrast required putting boundaries around the target sentence in PRAAT, and such a process could lead to some variability across trials and across untrained SLTs. As also suggested elsewhere [46], prosody should ideally be analyzed on local recordings instead of Zoom recordings, while not imperatively using professional quality recording hardware.

### 4.6. Limitations and Perspectives

In the present report, the mainstream software Zoom was used in 2020. However, algorithms used in online speech transmission are constantly evolving, and new features or updates have been added to Zoom since the collection of the present data (such as improved echo cancellation, noise reduction, automatic gain control, original sound preservation, etc.). It is also possible that other mass market software could provide audio transmission of similar or even better quality [47]. The same issue could be raised about all the hardware and the type of internet connection used in the present experiment, and future studies should investigate if the current results could be replicated for instance on tablet computers and with Voice over LTE technology. However, it will remain challenging to provide durable hardware recommendations to record speech at home [22], as the field is evolving extremely rapidly, with new hardware and software updates coming out incessantly.

Finally, future research should also investigate in larger samples the effect of demographic variables on online voice/speech samples analyses, as multi-dimensional variation, for instance, has been reported in adult speech as a function of age [48].

## 5. Conclusions

When teleassessing speech production, perceptual measures such as detecting errors in pseudoword reading/repeating and identifying words embedded in sentences without contextual cues do not seem comparable in online vs in-person recordings. In a hyperconnected world, speech intelligibility should henceforth be assessed not only in person, but also online. Several speech rate measures (number of words per second when repeating the days of the week, number of syllables per second in sequential and alternative motion rates) and mean f0 (in sentence reading) could be successfully computed on online recordings. To compute and interpret MPT and prosodic contrasts, in-person recordings with a built-in laptop microphone were of sufficient quality. As for the reliability of voice measurements, it still depended on high-quality in-person recordings.

## Figures and Tables

**Table 1:** Demographic information on the fifteen participants.

Participants	Age	Gender	Native Language (Area of Acquisition)	Country of Residence
P1	25	F	French (Ile de France)	France
P2	67	F	French (Jura)	Switzerland (French-speaking part)
P3	28	F	French (Auvergne-Rhône-Alpes)	France
P4	50	F	French (Ile de France)	France
P5	83	F	French (Auvergne-Rhône-Alpes)	France
P6	35	M	French (Fribourg)	Switzerland (French-speaking part)
P7	60	F	French (Fribourg)	Switzerland (French-speaking part)
P8	58	M	French (Auvergne-Rhône-Alpes)	France
P9	82	F	French (Neuchâtel)	Switzerland (French-speaking part)
P10	27	F	French (Auvergne-Rhône-Alpes)	France
P11	55	F	French (Auvergne-Rhône-Alpes)	France
P12	35	M	French (Fribourg)	Switzerland (French-speaking part)
P13	47	M	French (Auvergne-Rhône-Alpes)	France
P14	82	F	French (Fribourg)	Switzerland (French-speaking part)
P15	62	M	French (Fribourg)	Switzerland (French-speaking part)

**Table 2:** Key differences between the three recording conditions.

Condition	Recording Source	Origin of the Recorded Speech Signal	Recording Software	Audio Files Storage
Local HQ	Shure microphone and Scarlett external USB sound card connected to a Dell laptop situated next to the participant	In-person	MSD battery MonPaGe-2.0.s (sounddevice python library)	On the local computer
Local SQ	Built-in microphone of an Apple laptop situated in front of the participant	In-person	Safari browser (recorder js plugin)	On an online server
Online	Built-in microphone of an Apple laptop situated in front of the remote experimenter	Remote (played by the built-in speakers of the experimenter’s laptop via Zoom)	MSD battery MonPaGe-2.0.s (sounddevice python library)	On the remote computer

HQ = high-quality; SQ = standard quality.

**Table 3:** Mean raw differences (units in brackets), range of raw differences and mean ratio of divergence (%) between sources (with the local HQ condition serving as baseline reference).

			Intersource					
			Local HQ vs. Online			Local HQ vs. Local SQ		
			m	Range	%	m	Range	%
Acoustic measures	Speech rate	Rate_Sentence (syll/sec)	0.4	0–1.8	7.4	0.4	0–1.4	6.2
		Rate_Days (word/sec)	0	0–0.3	1.1	0	0–0.3	0.6
		Rate_DDK AMR CCV (syll/sec)	0.1	0–0.4	1.7	0	0–0.2	0.7
		Rate_DDK AMR CV (syll/sec)	0.1	0–0.2	1.4	0	0–0.1	0.4
		Rate_DDK SMR CV (syll/sec)	0.2	0–0.9	2.8	0	0–0.3	0.5
	Voice	CPPs /a/ (dB)	4.7	1.8–8.5	25	2.4	0.4–5.2	12.8
		HNR /a/ (dB)	6.3	2.2–12.9	29.9	3.5	0.2–10.1	16.3
		Jitter /a/ (%)	0.1	0–0.6	62.1	0.1	0–0.1	16.7
		SD f0 /a/ (Hz)	2.9	0–48.8	62.5	2.4	0–48.7	49.9
		Shimmer /a/ (%)	7.0	3.3–13.6	374.4	3.5	1.1–8.6	187.4
		CPPs sentence (dB)	4.7	1.8–7.8	25	2.4	0.4–5.1	13
		Mean f0 sentence (Hz)	5.6	0.3–22.2	2.8	3.4	0–14.3	1.7
		SD f0 sentence (Hz)	8.2	0.2–41.1	29.2	3.8	0–33.5	10.9
	Maximal Phonation Time (sec)		2.4	0–17.1	12.5	0.3	0–8.6	1.7
	Prosodic Contrast (semitones)		3.6	0–15.6	273.2	2.1	0–11.2	109.3
Perceptual measures	Articulation (errors in pseudowords)		1.3	0–7	0.9	0.6	0–5	0.4
	Intelligibility (incorrect words)		0.4	0–3	2.7	0.2	0–1	0.6
	Mean				53.8			25.2

m = raw differences averaged across participants and raters, range = range of the raw differences per participant, % = mean percentage of divergence.

**Table 4:** Mean raw differences (units in brackets), range of raw differences and mean ratio of divergence (%) between raters in each recording condition.

			Interrater								
			Local HQ			Local SQ			Online		
			m	Range	%	m	Range	%	m	Range	%
Acoustic measures	Speech rate	Rate_Sentence (syll/sec)	0.1	0–0.4	2.3	0.4	0.1–0.8	6.5	0.5	0–1.2	7.1
		Rate_Days (word/sec)	0	0–0.2	0.6	0	0–0.2	1.1	0	0–0.2	1.1
		Rate_DDK AMR CCV (syll/sec)	0	0–0.1	0.6	0	0–0.1	0.9	0	0–0.4	0.8
		Rate_DDK AMR CV (syll/sec)	0	0–0.2	0.5	0	0–0.2	0.6	0	0–0.2	0.5
		Rate_DDK SMR CV (syll/sec)	0	0–0.2	0.4	0	0–0.2	0.5	0.1	0–0.6	1.2
	Voice	CPPs /a/ (dB)	0	0–0.2	0.3	0.1	0–0.2	0.4	0.1	0–1.0	0.8
		HNR /a/ (dB)	0.3	0–2.2	1.0	0.2	0–0.8	0.8	0.1	0–0.4	1.4
		Jitter /a/ (%)	0	0–0.1	2.5	0	0–0.1	5.5	0	0–0.1	9.4
		SD f0 /a/ (Hz)	1.2	0–12.6	14.8	1.4	0–14.1	14.2	0.3	0–2.1	13.5
		Shimmer /a/ (%)	0	0–0.1	1.8	0.1	0–0.3	1.9	0.1	0–0.6	2.6
		CPPs sentence (dB)	0	0–0.2	0.3	0.1	0–0.2	0.4	0.1	0–0.3	0.5
		Mean f0 sentence (Hz)	2.1	0.2–8.7	0.8	0.6	0–5.9	0.5	2.6	0–8.4	1.2
		SD f0 sentence (Hz)	3.9	0.2–19.8	8.7	0.3	0–2.9	3.6	6.7	0–30.3	15.8
	Maximal Phonation Time (sec)		0.1	0–0.6	0.7	0.4	0–3.8	2.4	1.1	0–5.2	7.9
	Prosodic Contrast (semitones)		0.9	0.2–2.9	66.6	1.6	0.1–5.1	99.0	2.6	0.7–9.0	90.6
Perceptual measures	Articulation (errors in pseudowords)		0.1	0–1	0.1	0.8	0–5	0.3	1.1	0–5	0.4
	Intelligibility (incorrect words)		0.3	0–1	1.2	0.3	0–1	1.2	0.6	0–3	2.7
	Mean				6.1			8.2			9.3

m = raw differences averaged across participants and raters, range = range of the raw differences per participant, % = mean percentage of divergence.

**Table 5:** Intraclass Correlation Coefficients for all speech parameters.

			Intersource		Interrater		
			Local HQ vs. Online	Local HQ vs. Local SQ	Local HQ	Local SQ	Online
Acoustic measures	Speech rate	Rate_Sentence	0.87	0.89	0.97	0.77	0.77
		Rate_Days	0.99	0.99	0.98	0.96	0.96
		Rate_DDK AMR CCV	0.98	1	1	0.99	0.99
		Rate_DDK AMR CV	0.99	1	1	1	1
		Rate_DDK SMR CV	0.95	1	1	1	0.97
	Voice	CPPs /a/	0.38	0.72	1	1	0.99
		HNR /a/	0.08	0.17	0.90	0.99	0.99
		Jitter /a/	0.10	0.69	0.93	0.85	0.79
		SD f0 /a/	0.12	0.16	0.40	0.44	0.50
		Shimmer /a/	0.08	0.21	1	0.99	0.95
		CPPs sentence	0.38	0.72	1	1	1
		Mean f0 sentence	0.99	0.99	1	1	0.99
		SD f0 sentence	0.50	0.72	0.60	0.95	0.53
	Maximal Phonation Time		0.88	0.99	1	0.98	0.88
	Prosodic Contrast		0.43	0.76	0.79	0.60	0.43
Perceptual measures	Articulation (errors in pseudowords)		0.47	0.69	0.98	0.64	0.76
	Intelligibility (incorrect words)		0.27	0.44	0.46	0.13	0.39
	Mean ICC		0.56	0.70	0.91	0.84	0.82

## Data Availability

The raw data is available in the Supplementary Materials.

## Supplementary Materials

The following supporting information can be downloaded at: https://www.mdpi.com/article/10.3390/brainsci13020342/s1.
